# 4-(4-Pyrid­yl)pyridinium perchlorate methanol solvate

**DOI:** 10.1107/S1600536810012985

**Published:** 2010-04-14

**Authors:** Yu-Hua Gao, De-Ming Wu

**Affiliations:** a School of Materials Science and Engineering, Jiangsu University of Science and Technology, Zhenjiang 212003, People’s Republic of China

## Abstract

In the cation of the title hydrated molecular salt, C_10_H_9_N_2_^+^·ClO_4_^−^·CH_3_OH, the dihedral angle formed by the pyridine rings is 28.82 (15)°. The crystal structure is stabilized by inter­molecular N—H⋯O and O—H⋯N hydrogen bonds and π–π stacking inter­actions, with centroid-to-centroid distances of 3.5913 (7) and 3.6526 (7) Å. Three O atoms of the perchlorate anion are disordered over two positions with refined occupancy factors of 0.649 (7):0.351 (7).

## Related literature

For simple mol­ecular–ionic crystals containing organic cations and acid radicals, see: Katrusiak & Szafrański (1999 ▶, 2006 ▶). For the crystal structure of 4,4′-bipyridin-1-ium perchlorate di­hydrate, see: Zhang *et al.* (2008 ▶).
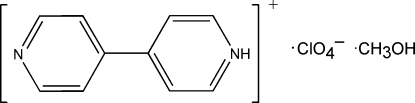


## Experimental

### 

#### Crystal data

C_10_H_9_N_2_^+^·ClO_4_^−^·CH_4_O
*M* *_r_* = 288.68
Monoclinic, 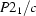

*a* = 6.8822 (14) Å
*b* = 15.362 (3) Å
*c* = 12.254 (3) Å
β = 92.07 (3)°
*V* = 1294.7 (5) Å^3^
*Z* = 4
Mo *K*α radiation
μ = 0.31 mm^−1^
*T* = 293 K
0.3 × 0.26 × 0.2 mm

#### Data collection

Rigaku SCXmini diffractometer
Absorption correction: multi-scan (*CrystalClear*; Rigaku, 2005 ▶) *T*_min_ = 0.62, *T*_max_ = 0.81
13295 measured reflections
2956 independent reflections
1803 reflections with *I* > 2σ(*I*)
*R*_int_ = 0.067

#### Refinement

*R*[*F* ^2^ > 2σ(*F* ^2^)] = 0.079
*wR*(*F* ^2^) = 0.210
*S* = 1.00
2956 reflections
201 parameters
88 restraints
H-atom parameters constrained
Δρ_max_ = 0.63 e Å^−3^
Δρ_min_ = −0.60 e Å^−3^

### 

Data collection: *CrystalClear* (Rigaku, 2005 ▶); cell refinement: *CrystalClear*; data reduction: *CrystalClear*; program(s) used to solve structure: *SHELXS97* (Sheldrick, 2008 ▶); program(s) used to refine structure: *SHELXL97* (Sheldrick, 2008 ▶); molecular graphics: *SHELXTL* (Sheldrick, 2008 ▶); software used to prepare material for publication: *SHELXL97*.

## Supplementary Material

Crystal structure: contains datablocks I, global. DOI: 10.1107/S1600536810012985/rz2430sup1.cif

Structure factors: contains datablocks I. DOI: 10.1107/S1600536810012985/rz2430Isup2.hkl

Additional supplementary materials:  crystallographic information; 3D view; checkCIF report

## Figures and Tables

**Table 1:** Hydrogen-bond geometry (Å, °)

*D*—H⋯*A*	*D*—H	H⋯*A*	*D*⋯*A*	*D*—H⋯*A*
O5—H5⋯N2^i^	0.88	1.99	2.857 (5)	167
N1—H1*B*⋯O5^ii^	0.86	2.12	2.825 (5)	139
N1—H1*B*⋯O1^iii^	0.86	2.31	3.010 (5)	138

Symmetry codes: (i) 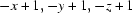; (ii) 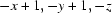; (iii) 
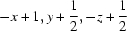
.

**Fig. 1.**
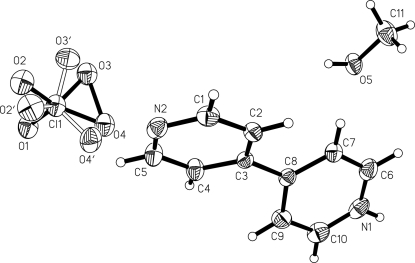
The asymmetric unit of the title compound with atom labels. Displacement ellipsoids were drawn at the 30% probability level.

**Fig. 2.**
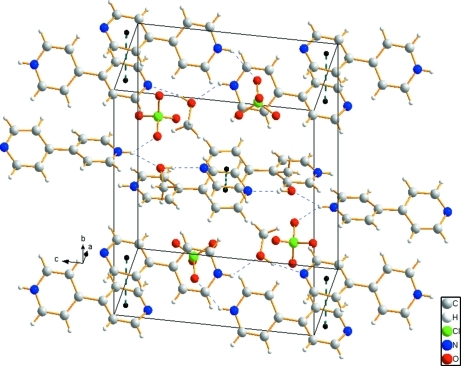
Packing diagram of the title compound. Intermolecular hydrogen bonds and centroid-to-centroid distances are drawn as dashed lines.

## supplementary crystallographic information

### Comment

Recently, much attention has been devoted to simple molecular–ionic crystals containing organic cations and acid radicals in 1:1 molar ratio due to the tunability of their special structural features and their interesting physical properties (Katrusiak & Szafrański, 1999, 2006). The crystal structure of 4,4'-bipyridin-1-ium perchlorate dihydrate have been reported (Zhang *et al.*, 2008). In our laboratory, a compound containing a protonated 4,4'-bipyridin-1-ium cation has been synthesized, and its crystal structure is reported herein.

The asymmetric unit of the title compound (Fig. 1) consists of one 4,4'-bipyridin-1-ium cation, one ClO_4_^-^ anion and one methanol molecule. In the cation, the pyridine rings are tilted by 28.82 (15)°. The crystal structure is stabilized by intermolecular N—H···O and O—H···N hydrogen bonds (Table 1) and π–π stacking interactions involving the unprotonated pyridine rings, with centroid-to-centroid distances of 3.5913 (7) and 3.6526 (7) Å. The hydrogen bonds result in the formation of chains along the *c* axis (Fig. 2).

### Experimental

4,4'-Bipyridine (10 mmol) and 10% aqueous HClO_4_ in a molar ratio of 1:1 were mixed and dissolved in methanol. The mixture was heated to 323 K until a clear solution formed. The reaction mixture was cooled slowly to room temperature, crystals of the title compound suitable for X-ray analysis were obtained, collected and washed with dilute aqueous HClO_4_.

### Refinement

All H atoms were placed in calculated positions, with C—H = 0.93 Å, O—H = 0.85 Å and N—H = 0.86 Å, and refined using a riding model, with U_iso_(H) = 1.2U_eq_(C, N) or 1.5U_eq_(O). The O2, O3 and O4 oxygen atoms of the perchlorate anion are disordered over two positions with refined occupancy factors of 0.649 (7):0.351 (7). Within the anion, the geometry of the Cl—O bonds was restrained to be similar by the *SAME* instruction, and the displacement ellipsoids were restrained to be nearly isotropic by the *ISOR* instruction.

### Crystal data

**Table:**

C_10_H_9_N_2_^+^·ClO_4_^−^·CH_4_O	*F*(000) = 600
*M**_r_* = 288.68	*D*_x_ = 1.481 Mg m^−^^3^
Monoclinic, *P*2_1_/*c*	Mo *K*α radiation, λ = 0.71073 Å
Hall symbol: -P 2ybc	Cell parameters from 1803 reflections
*a* = 6.8822 (14) Å	θ = 3.1–27.5°
*b* = 15.362 (3) Å	µ = 0.31 mm^−^^1^
*c* = 12.254 (3) Å	*T* = 293 K
β = 92.07 (3)°	Block, colourless
*V* = 1294.7 (5) Å^3^	0.3 × 0.26 × 0.2 mm
*Z* = 4	

### Data collection

**Table:**

Rigaku SCXmini diffractometer	2956 independent reflections
Radiation source: fine-focus sealed tube	1803 reflections with *I* > 2σ(*I*)
graphite	*R*_int_ = 0.067
Detector resolution: 13.6612 pixels mm^-1^	θ_max_ = 27.5°, θ_min_ = 3.1°
ω scans	*h* = −8→8
Absorption correction: multi-scan (*CrystalClear*; Rigaku, 2005)	*k* = −19→19
*T*_min_ = 0.62, *T*_max_ = 0.81	*l* = −15→15
13295 measured reflections	

### Refinement

**Table:**

Refinement on *F*^2^	Primary atom site location: structure-invariant direct methods
Least-squares matrix: full	Secondary atom site location: difference Fourier map
*R*[*F*^2^ > 2σ(*F*^2^)] = 0.079	Hydrogen site location: inferred from neighbouring sites
*wR*(*F*^2^) = 0.210	H-atom parameters constrained
*S* = 1.00	*w* = 1/[σ^2^(*F*_o_^2^) + (0.0767*P*)^2^ + 2.5927*P*] where *P* = (*F*_o_^2^ + 2*F*_c_^2^)/3
2956 reflections	(Δ/σ)_max_ < 0.001
201 parameters	Δρ_max_ = 0.63 e Å^−^^3^
88 restraints	Δρ_min_ = −0.60 e Å^−^^3^

### Special details

**Table:**

Geometry. All esds (except the esd in the dihedral angle between two l.s. planes) are estimated using the full covariance matrix. The cell esds are taken into account individually in the estimation of esds in distances, angles and torsion angles; correlations between esds in cell parameters are only used when they are defined by crystal symmetry. An approximate (isotropic) treatment of cell esds is used for estimating esds involving l.s. planes.
Refinement. Refinement of *F*^2^ against ALL reflections. The weighted *R*-factor wR and goodness of fit *S* are based on *F*^2^, conventional *R*-factors *R* are based on *F*, with *F* set to zero for negative *F*^2^. The threshold expression of *F*^2^ > σ(*F*^2^) is used only for calculating *R*-factors(gt) etc. and is not relevant to the choice of reflections for refinement. *R*-factors based on *F*^2^ are statistically about twice as large as those based on *F*, and *R*- factors based on ALL data will be even larger.

### Fractional atomic coordinates and isotropic or equivalent isotropic displacement parameters (Å^2^)

**Table:**

	*x*	*y*	*z*	*U*_iso_*/*U*_eq_	Occ. (<1)
C1	0.7627 (6)	0.5390 (3)	0.5916 (4)	0.0459 (11)	
H1A	0.7704	0.5882	0.6356	0.055*	
C2	0.7575 (6)	0.5504 (3)	0.4801 (3)	0.0395 (10)	
H2A	0.7624	0.6061	0.4505	0.047*	
C3	0.7449 (6)	0.4786 (3)	0.4128 (3)	0.0348 (9)	
C4	0.7326 (7)	0.3979 (3)	0.4625 (4)	0.0464 (11)	
H4A	0.7185	0.3478	0.4205	0.056*	
C5	0.7414 (7)	0.3929 (3)	0.5742 (4)	0.0522 (12)	
H5A	0.7360	0.3380	0.6060	0.063*	
C6	0.6765 (7)	0.5634 (4)	0.1258 (4)	0.0564 (13)	
H6A	0.6278	0.6121	0.0889	0.068*	
C7	0.6747 (7)	0.5599 (3)	0.2371 (4)	0.0469 (11)	
H7A	0.6270	0.6067	0.2763	0.056*	
C8	0.7450 (6)	0.4859 (3)	0.2922 (3)	0.0370 (9)	
C9	0.8198 (7)	0.4193 (3)	0.2298 (4)	0.0494 (11)	
H9A	0.8702	0.3696	0.2637	0.059*	
C10	0.8194 (8)	0.4267 (4)	0.1186 (4)	0.0565 (13)	
H10A	0.8698	0.3820	0.0769	0.068*	
C11	0.1539 (10)	0.6691 (3)	0.1471 (5)	0.0727 (17)	
H11A	0.0837	0.6922	0.0844	0.087*	
H11B	0.2789	0.6967	0.1541	0.087*	
H11C	0.0823	0.6801	0.2114	0.087*	
N1	0.7476 (6)	0.4972 (3)	0.0702 (3)	0.0547 (11)	
H1B	0.7470	0.5003	0.0002	0.066*	
N2	0.7572 (5)	0.4616 (3)	0.6398 (3)	0.0472 (9)	
O5	0.1785 (5)	0.5786 (2)	0.1341 (2)	0.0537 (9)	
H5	0.2107	0.5599	0.2002	0.081*	
Cl1	0.32539 (19)	0.19524 (7)	0.64038 (10)	0.0531 (4)	
O1	0.3132 (6)	0.1030 (2)	0.6349 (3)	0.0634 (9)	
O2	0.335 (2)	0.2182 (8)	0.7540 (9)	0.0659 (15)	0.351 (7)
O3	0.1581 (17)	0.2295 (7)	0.5822 (11)	0.0606 (9)	0.351 (7)
O4	0.3801 (19)	0.2310 (7)	0.5350 (9)	0.0601 (9)	0.351 (7)
O2'	0.4433 (12)	0.2213 (4)	0.7329 (6)	0.0739 (13)	0.649 (7)
O3'	0.1388 (10)	0.2337 (4)	0.6422 (7)	0.0717 (12)	0.649 (7)
O4'	0.4702 (11)	0.2228 (4)	0.5685 (6)	0.0732 (12)	0.649 (7)

### Atomic displacement parameters (Å^2^)

**Table:**

	*U*^11^	*U*^22^	*U*^33^	*U*^12^	*U*^13^	*U*^23^
C1	0.046 (3)	0.048 (3)	0.044 (3)	0.001 (2)	0.000 (2)	−0.008 (2)
C2	0.042 (2)	0.036 (2)	0.040 (2)	0.0015 (18)	−0.0002 (18)	0.0001 (17)
C3	0.032 (2)	0.039 (2)	0.034 (2)	0.0004 (17)	0.0020 (16)	0.0025 (17)
C4	0.062 (3)	0.036 (2)	0.041 (2)	−0.005 (2)	−0.001 (2)	−0.0002 (18)
C5	0.065 (3)	0.049 (3)	0.042 (2)	−0.004 (2)	0.003 (2)	0.009 (2)
C6	0.050 (3)	0.073 (3)	0.046 (3)	−0.002 (3)	−0.001 (2)	0.018 (3)
C7	0.049 (3)	0.050 (3)	0.042 (2)	0.003 (2)	0.004 (2)	0.008 (2)
C8	0.034 (2)	0.043 (2)	0.033 (2)	−0.0030 (18)	0.0007 (17)	0.0013 (17)
C9	0.055 (3)	0.051 (3)	0.042 (3)	0.005 (2)	0.003 (2)	−0.005 (2)
C10	0.059 (3)	0.073 (4)	0.038 (3)	−0.002 (3)	0.002 (2)	−0.011 (2)
C11	0.110 (5)	0.051 (3)	0.056 (3)	0.008 (3)	−0.010 (3)	−0.003 (3)
N1	0.043 (2)	0.089 (3)	0.0322 (19)	−0.011 (2)	−0.0008 (17)	0.004 (2)
N2	0.047 (2)	0.059 (2)	0.0356 (19)	−0.0010 (18)	0.0017 (16)	0.0006 (18)
O5	0.082 (2)	0.0453 (18)	0.0341 (16)	0.0012 (16)	−0.0031 (15)	0.0001 (13)
Cl1	0.0674 (8)	0.0371 (6)	0.0546 (7)	−0.0098 (5)	0.0003 (5)	−0.0051 (5)
O1	0.093 (2)	0.0369 (12)	0.0603 (19)	−0.0094 (12)	0.0059 (17)	−0.0059 (12)
O2	0.084 (3)	0.055 (3)	0.0592 (16)	−0.012 (3)	0.000 (2)	−0.015 (2)
O3	0.0715 (16)	0.0479 (16)	0.0622 (17)	−0.0064 (14)	−0.0005 (14)	−0.0015 (16)
O4	0.0722 (17)	0.0470 (16)	0.0609 (15)	−0.0095 (16)	0.0001 (15)	0.0018 (14)
O2'	0.084 (3)	0.063 (2)	0.074 (2)	−0.014 (3)	−0.010 (2)	−0.019 (2)
O3'	0.0757 (18)	0.062 (2)	0.077 (3)	0.0039 (18)	0.004 (2)	−0.011 (2)
O4'	0.074 (2)	0.066 (2)	0.080 (2)	−0.017 (2)	0.007 (2)	0.012 (2)

### Geometric parameters (Å, °)

**Table:**

C1—N2	1.329 (6)	C9—H9A	0.9300
C1—C2	1.376 (6)	C10—N1	1.322 (7)
C1—H1A	0.9300	C10—H10A	0.9300
C2—C3	1.379 (6)	C11—O5	1.411 (6)
C2—H2A	0.9300	C11—H11A	0.9600
C3—C4	1.384 (6)	C11—H11B	0.9600
C3—C8	1.481 (5)	C11—H11C	0.9600
C4—C5	1.370 (6)	N1—H1B	0.8600
C4—H4A	0.9300	O5—H5	0.8804
C5—N2	1.329 (6)	Cl1—O3'	1.415 (7)
C5—H5A	0.9300	Cl1—O4'	1.419 (6)
C6—N1	1.327 (7)	Cl1—O1	1.420 (3)
C6—C7	1.366 (7)	Cl1—O2'	1.428 (6)
C6—H6A	0.9300	Cl1—O3	1.432 (12)
C7—C8	1.400 (6)	Cl1—O2	1.435 (11)
C7—H7A	0.9300	Cl1—O4	1.465 (11)
C8—C9	1.387 (6)	O3—O4	1.653 (19)
C9—C10	1.368 (6)		
			
N2—C1—C2	123.8 (4)	O5—C11—H11B	109.5
N2—C1—H1A	118.1	H11A—C11—H11B	109.5
C2—C1—H1A	118.1	O5—C11—H11C	109.5
C1—C2—C3	119.3 (4)	H11A—C11—H11C	109.5
C1—C2—H2A	120.3	H11B—C11—H11C	109.5
C3—C2—H2A	120.3	C10—N1—C6	122.5 (4)
C2—C3—C4	117.2 (4)	C10—N1—H1B	118.8
C2—C3—C8	122.3 (4)	C6—N1—H1B	118.8
C4—C3—C8	120.6 (4)	C1—N2—C5	116.4 (4)
C5—C4—C3	119.3 (4)	C11—O5—H5	104.2
C5—C4—H4A	120.4	O3'—Cl1—O4'	122.9 (5)
C3—C4—H4A	120.4	O3'—Cl1—O1	111.4 (3)
N2—C5—C4	124.0 (4)	O4'—Cl1—O1	108.1 (3)
N2—C5—H5A	118.0	O3'—Cl1—O2'	111.1 (4)
C4—C5—H5A	118.0	O4'—Cl1—O2'	91.0 (6)
N1—C6—C7	120.0 (5)	O1—Cl1—O2'	110.4 (3)
N1—C6—H6A	120.0	O4'—Cl1—O3	98.7 (7)
C7—C6—H6A	120.0	O1—Cl1—O3	107.3 (5)
C6—C7—C8	119.9 (5)	O2'—Cl1—O3	135.7 (6)
C6—C7—H7A	120.1	O3'—Cl1—O2	83.7 (6)
C8—C7—H7A	120.1	O4'—Cl1—O2	121.4 (7)
C9—C8—C7	117.5 (4)	O1—Cl1—O2	106.9 (5)
C9—C8—C3	120.5 (4)	O3—Cl1—O2	113.5 (7)
C7—C8—C3	122.0 (4)	O3'—Cl1—O4	96.9 (7)
C10—C9—C8	120.0 (5)	O1—Cl1—O4	110.4 (5)
C10—C9—H9A	120.0	O2'—Cl1—O4	116.0 (7)
C8—C9—H9A	120.0	O3—Cl1—O4	69.6 (8)
N1—C10—C9	120.1 (5)	O2—Cl1—O4	139.4 (7)
N1—C10—H10A	119.9	Cl1—O3—O4	56.1 (6)
C9—C10—H10A	119.9	Cl1—O4—O3	54.3 (6)
O5—C11—H11A	109.5		

### Hydrogen-bond geometry (Å, °)

**Table:**

*D*—H···*A*	*D*—H	H···*A*	*D*···*A*	*D*—H···*A*
O5—H5···N2^i^	0.88	1.99	2.857 (5)	167
N1—H1B···O5^ii^	0.86	2.12	2.825 (5)	139
N1—H1B···O1^iii^	0.86	2.31	3.010 (5)	138

Symmetry codes: (i) −*x*+1, −*y*+1, −*z*+1; (ii) −*x*+1, −*y*+1, −*z*; (iii) −*x*+1, *y*+1/2, −*z*+1/2.

## References

[bb1] Katrusiak, A. & Szafrański, M. (1999). *Phys. Rev. Lett.* **82**, 576–579.

[bb2] Katrusiak, A. & Szafrański, M. (2006). *J. Am. Chem. Soc.* **128**, 15775–15785. 10.1021/ja0650192 17147387

[bb3] Rigaku (2005). *CrystalClear* Rigaku Corporation, Tokyo, Japan.

[bb4] Sheldrick, G. M. (2008). *Acta Cryst.* A**64**, 112–122. 10.1107/S0108767307043930 18156677

[bb5] Zhang, J.-Y., Chen, A.-L. & Gao, E.-Q. (2008). *J. Chem. Crystallogr.* **38**, 351–355.

